# Utilization of High-Flow Nasal Cannulas in Pediatric Critical Care: A Single-Center Experience

**DOI:** 10.7759/cureus.75742

**Published:** 2024-12-15

**Authors:** Ahmed M Omran, Sawsan M AIYousef, Jihad A Zahraa, Muath S AlMubarak, Muath S Albalawi, Reema F Alshenaifi, Ohood A AlRohaili, Raed N Sadek

**Affiliations:** 1 Pediatric Intensive Care Unit, King Fahad Medical City, Riyadh, SAU; 2 Intensive Care Unit, AI Jalila Children’s Specialty Hospital, Dubai, ARE; 3 Pediatric Infectious Diseases, King Fahad Medical City, Riyadh, SAU; 4 Pediatric Emergency, King Fahad Medical City, Riyadh, SAU; 5 Pediatric Emergency, Medical Cities Program, Ministry of Interior (MOI), Riyadh, SAU

**Keywords:** hfnc success/failure rates, high-flow nasal cannula (hfnc), non-invasive ventilation (niv), pediatric intensive care unit (picu), respiratory distress/failure

## Abstract

Background: High-flow nasal cannula (HFNC) therapy has developed as a valuable tool for respiratory support in pediatric critical care. It offers an intermediate level of support between traditional low-flow oxygen and non-invasive ventilation (NIV). Studies suggest its effectiveness in improving oxygen delivery, work of breathing, and secretion clearance.

Method: This retrospective study reviewed medical records of 265 pediatric patients aged between one day to 14 years admitted to the pediatric intensive care unit (PICU) for respiratory distress between 2014 and 2019, who required HFNC therapy. Analyzed data on demographics, diagnoses, HFNC settings, physiologic parameters, complications, and outcomes (e.g., escalation of support, length of stay) using IBM SPSS Statistics software, version 23 (IBM Corp., Armonk, NY).

Results: Age, indication, and positive viral nasopharyngeal swabs (NPA) didn't influence the outcome of HFNC. The most common primary admission diagnosis was pneumonia (38.5%); other indications for HFNC treatment included septic shock, pleural effusion, and upper airway obstruction. However, low durations of HFNC, elevated carbon dioxide (CO2), no improvement in potential of hydrogen (pH) after initiation of therapy, and elevated respiratory rate were observed more in the failure group.

Conclusion: High-flow nasal cannula therapy emerges as a valuable alternative to invasive ventilation in PICU patients. Blood gases, respiratory rate, and heart rate (HR) are important parameters, besides clinical examination, to closely monitor the children susceptible to failure.

## Introduction

High-flow nasal cannula (HFNC) therapy has emerged as a game-changer in respiratory management. Delivering warmed and humidified gas at high flow rates, HFNC therapy bridges the gap between traditional low-flow oxygen and non-invasive ventilation (NIV) techniques like continuous positive airway pressure (CPAP) or bi-level positive airway pressure (BiPAP). This intermediate level of support offers potential benefits such as improved work of breathing, increased oxygen delivery, and enhanced secretion clearance [1].

The use of HFNC therapy has skyrocketed across various patient populations, finding applications in critically ill neonates, infants, children, and even adults. Compared to standard nasal cannulas or facemasks, HFNCs appear to provide a more comfortable and effective level of respiratory support. This has led to its wider acceptance in diverse healthcare settings, including neonatal intensive care units (NICUs), pediatric intensive care units (PICUs), intermediate care units, emergency departments (EDs), and pediatric general wards [2].

Clinical trials have yielded promising results for HFNC therapy, particularly in pediatrics. A landmark study by Eşki et al. demonstrated the potential superiority of HFNCs to standard low-flow oxygen in preventing treatment failure for children with bronchiolitis [3]. This study, along with others, suggests that HFNCs can be an effective bridge therapy, potentially delaying or even avoiding the need for escalation to NIV [4].

This study contributed to the growing body of knowledge on HFNC therapy in the PICU setting. By understanding the effectiveness and limitations of HFNCs in our specific patient population, we were able to optimize its use and potentially improve patient outcomes while reducing the need for more invasive respiratory support techniques.

## Materials and methods

This study aimed to address knowledge gaps regarding the use of HFNC therapy in PICU. By analyzing a retrospective cohort of patients who received HFNC therapy, we hoped to gain insights into the prevalence of HFNC use in various etiologies of acute respiratory distress and/or failure, the clinical and gas exchange responses to HFNC therapy, the efficacy of HFNCs in avoiding more aggressive respiratory support, the factors associated with success versus failure of HFNC therapy, and the potential complications associated with HFNC use in infants and children.

A retrospective, descriptive cohort design was employed to investigate these objectives. Using virtual patient system (VPS) data, a convenience sample of 265 patients admitted to the PICU of King Fahad Medical City (KFMC), Riyadh, Saudi Arabia, between 2014 and 2019 who received HFNC therapy was identified. Detailed information was then extracted from patients' medical records through the KFMC semi-electronic health information system (Health Information Management (HIM) viewer developed for KFMC by NVSSoft®, Riyadh, Saudi Arabia).

The inclusion criteria for the study were patients aged between one day to 14 years who were admitted to the PICU between 2014 and 2019 and experienced respiratory distress/failure requiring a higher level of support than traditional oxygen delivery devices. Patients were excluded if they failed HFNC therapy and required escalation of support within one hour of initiation, experienced deterioration due to unrelated organ dysfunction, or had incomplete medical record data.

A standardized data collection form was used to record patient information. The primary investigator and two co-investigators reviewed medical records to extract data on demographics, underlying diseases, primary diagnosis, HFNC duration, indications for HFNC, severity of illness, vital signs, fraction of inspired oxygen (FiO_^2^_), HFNC flow rate, blood gas values, starting and maximum HFNC settings, complications, mode of respiratory support escalation, and length of PICU and hospital stays.

Before commencing the study, the Institutional Review Board (IRB) of KFMC granted ethical approval (approval number Federal Wide Assurance NIH, USA: FWA00018774).

Following data collection, the data were coded and entered into a statistical software program (IBM SPSS Statistics software, version 23 (IBM Corp., Armonk, NY)). Statistical analysis was conducted using appropriate methods based on data distribution. The Shapiro-Wilks test was applied to check the data distribution. The independent t-test was used to study statistical differences in the normally distributed data between groups. However, Mann-Whitney and chi-square tests were applied to compare non-normally distributed or categorical variables between groups.

## Results

This study included a total of 265 patients treated with HFNC. There were no statistically significant differences in age, gender, or nationality between the two groups (success group and failure group). The median age across all patients was nine months; males comprised slightly more than half the study population (61.1%), with females accounting for the remaining (38.9%) (Table 1). The vast majority of patients (93.2%) were Saudi nationals. Body mass index (BMI) was also comparable between the two groups, with a median value of 14.60 kg/m² (interquartile range: 12.20-17.10 kg/m²). Notably, the duration of HFNC treatment differed significantly between the groups (p < 0.001). Patients successfully weaned from HFNCs required the device for a median duration of 28 hours (interquartile range: 15-55 hours), whereas those who experienced failure had a median duration of 19 hours (interquartile range: 8-34.50 hours).

**Table 1:** Comparison of the main demographic information and comorbidities between patients weaned off HFNC and those who experienced HFNC failure and required intervention P < 0.05 represents a significant difference between success and failure groups. ^a,b^: Each letter denotes a subset of the type of diagnosis. Proportions marked with different letters are significantly different (p<0.05). Data are displayed as n (%) or median (interquartile range). HFNC: high-flow nasal cannula; BMI: body mass index; viral NPA test: viral nasopharyngeal aspirate (NPA) test; RSV: respiratory syncytial virus; MRSA: methicillin-resistant *Staphylococcus aureus*; Paraflu: human parainfluenza viruses

Parameter	Total (n=265)	HNFC outcome		p-value
		Success (n=184)	Failure (n=81)	
Demographics, body mass index, and HFNC duration				
Age (months)	9 (3-30)	9 (3-28)	10 (4-36)	0.600
Gender (male)	162 (61.1%)	117 (63.6%)	45 (55.6%)	0.136
Gender (female)	103 (38.9%)	67 (36.4%)	36 (44.4%)	
Nationality (Saudi)	247 (93.2%)	171 (92.9%)	76 (93.8%)	0.512
Nationality (non-Saudi)	18 (6.8%)	13 (7.1%)	5 (6.2%)	
BMI (kg/m^2^)	14.60 (12.20- 17.10)	14.30 (12.13- 17.35)	14.90 (12.20- 16.90)	0.587
HFNC duration (hours)	28 (15.00- 55.00)	36.50 (19.00- 67.50)	19 (8.00-34.50)	0.001
Indications				
Pneumonia	1 (0.4%)	0.0 (0.0%)^a^	1 (0.5%)^a^	0.033
Hypercarpea	4 (1.5%)	3 (1.6%)^a^	1 (1.3%)^a^	
Hypoxemia	7 (2.7%)	5 (2.7%)^a^	2 (2.5%)^a^	
Post extubation	9 (3.4%)	7 (3.8%)^a^	2 (2.5%)^a^	
Apnea	12 (4.5%)	11 (6.0%)^a^	1 (1.3%)^a^	
Respiratory distress	30 (11.4%)	16 (8.7%)^b^	14 (17.5%)^a^	
High work of breathing (WOB)	58 (22%)	49 (26.6%)^b^	9 (11.3%)^a^	
Combined	143 (54.2%)	92 (50%)^b^	51 (63.8%)^a^	
Underlying diseases				
Positive	35 (13.2%)	25 (13.6%)	10 (12.3%)	0.477
Negative	230 (86.8%)	159 (86.4%)	71 (87.7%)	
HFNC complications				
Yes	8 (3.0%)	0 (0.0%)	8 (9.9%)	0.001
No	257 (97%)	184 (100%)	73 (90.1%)	
Viral NPA test				
Negative	117 (44.2%)	80 (43.5%)	37 (45.7%)	0.649
RSV	32 (12.1%)	23 (12.5%)	9 (11.1%)	
Adeno	12 (4.5%)	7 (3.8%)	5 (6.2%)	
Paraflu	4 (1.5%)	4 (2.2%)	0 (0.0%)	
Rhino	46 (17.4%)	31 (16.8%)	15 (18.5%)	
Influenza A	8 (3.0%)	4 (2.2%)	4 (4.9%)	
Influenza B	1 (0.4%)	1 (0.5%)	0 (0.0%)	
Metapneumo	2 (0.8%)	1 (0.5%)	1 (1.2%)	
COVID-19	4 (1.5%)	2 (1.1%)	2 (2.5%)	
Combined	35 (13.2%)	28 (15.2%)	7 (8.6%)	
Bocavirus	2 (0.8%)	2 (1.1%)	0 (0.0%)	
MRSA	2 (0.8%)	1 (0.5%)	1 (1.2%)	

Regarding the viral NPA, the positive rate of pathogens was 55.8%, and the most frequent virus found was rhinovirus. The presence or absence of viruses had nothing to do with the outcome of HFNC therapy. The use of HFNCs was required when the patient had deteriorated, showing signs of distress such as hypercapnia, hypoxemia, tachypnea, high work of breathing, hemodynamic instability, or respiratory distress. However, in cases with respiratory distress or multiple combined indications, the percentage of failure was markedly greater than in the success group (Table 1).

The most common primary admission diagnosis was pneumonia (38.5%), followed by bronchiolitis (13.2%), and then bronchial asthma was present in 8.7% of cases (Table 2). In our study, most of the patients showed improvement with HFNC therapy (69.4%); cases that showed no improvement with HFNCs were either treated with intubation (17% of cases) or NIV (13.6% of cases). The highest percentage of successful groups was weaned to a nasal cannula (67.1% of cases).

**Table 2:** A comparison of the frequency of various diagnoses and outcomes between patients who successfully weaned from HFNC and those who required further intervention. P < 0.05 represents a significant difference between success and failure groups. HFNC: high-flow nasal cannula; UAO: upper airway obstruction; ACS: acute coronary syndrome; pertussis: *Bordetella pertussis* bacteria; NIV: non-invasive ventilation; NC: nasal cannula; FM: face mask; NRM: non-rebreather mask; RA: room air

Parameter	Total (n=265)	HNFC outcome		p-value
		Success (n=184)	Failure (n=81)	
Admission diagnosis				
Pneumonia	102 (38.5%)	72 (39.1%)	30 (37.0%)	0.055
Septic shock	20 (7.5%)	9 (4.9%)	11 (13.6%)	
Pleural effusion	4 (1.5%)	2 (1.1%)	2 (2.5%)	
Bronchiolitis	35 (13.2%)	29 (15.8%)	6 (7.4%)	
Asthma	23 (8.7%)	17 (9.2%)	6 (7.4%)	
UAO	4 (1.5%)	3 (1.6%)	1 (1.2%)	
ACS	2 (0.8%)	2 (1.1%)	0 (0.0%)	
Pulmonary edema	2 (0.8%)	0 (0.0%)	2 (2.5%)	
Pertussis	1 (0.4%)	1 (0.5%)	0 (0.0%)	
Lung collapse	3 (1.1%)	2 (1.1%)	1 (1.2%)	
Laryngotracheitis	1 (0.4%)	1 (0.5%)	0 (0.0%)	
Apnea	22 (8.3%)	18 (9.8%)	4 (4.9%)	
Cardiogenic shock	2 (0.8%)	1 (0.5%)	1 (1.2%)	
Combined	18 (6.8%)	8 (4.3%)	10 (12.3%)	
Others	26 (9.8%)	19 (10.3%)	7 (8.6%)	
Outcome				
Intubation	45 (17.0%)	0(0.0%)	45(55.6%)	0.001
NIV	36 (13.6%)	0(0.0%)	36(44.4%)	
Weaned to				
NC	173 (67.1%)	173 (94.0%)	0 (0.0%)	0.001
FM	4 (1.6%)	4 (2.2%)	0 (0.0%)	
NRM	2 (0.8%)	2 (1.1%)	0 (0.0%)	
SRM	1 (0.4%)	1 (0.5%)	0 (0.0%)	
RA	4 (1.6%)	4 (2.2%)	0 (0.0%)	

**Figure 1:**
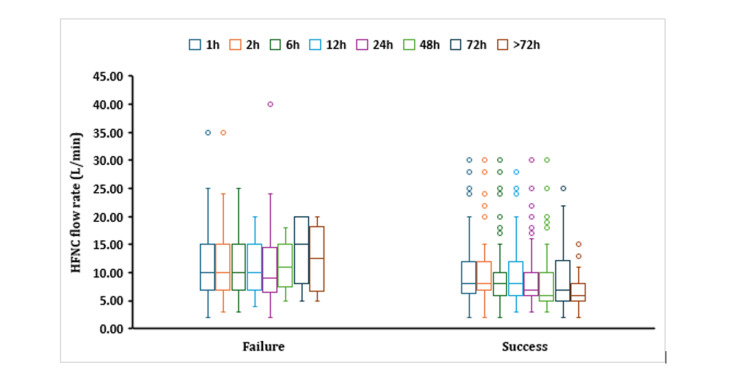
Box plot showing median HFNC flow rate for children who were on HFNC against the outcome of HFNC at different time intervals. HFNC: high-flow nasal cannula; h: hours

Only eight cases experienced complications, mostly due to nasal irritation, with no observed major complications. Regarding the flow rate of HFNCs, in the success group, it was mostly lower than in the failure group, especially after six, 12, 24, 48, and 72 hours of using HFNC (Figure 1).

The respiratory oxygenation (ROX) index in the PICU is a clinical tool used to assess the success of HFNC therapy in children with respiratory distress. It is calculated by dividing the ratio of oxygen saturation (SpO₂) to FiO₂ by the respiratory rate. This index helps clinicians predict whether a patient is likely to respond well to HFNC therapy or may require escalation to more invasive respiratory support.

**Figure 2:**
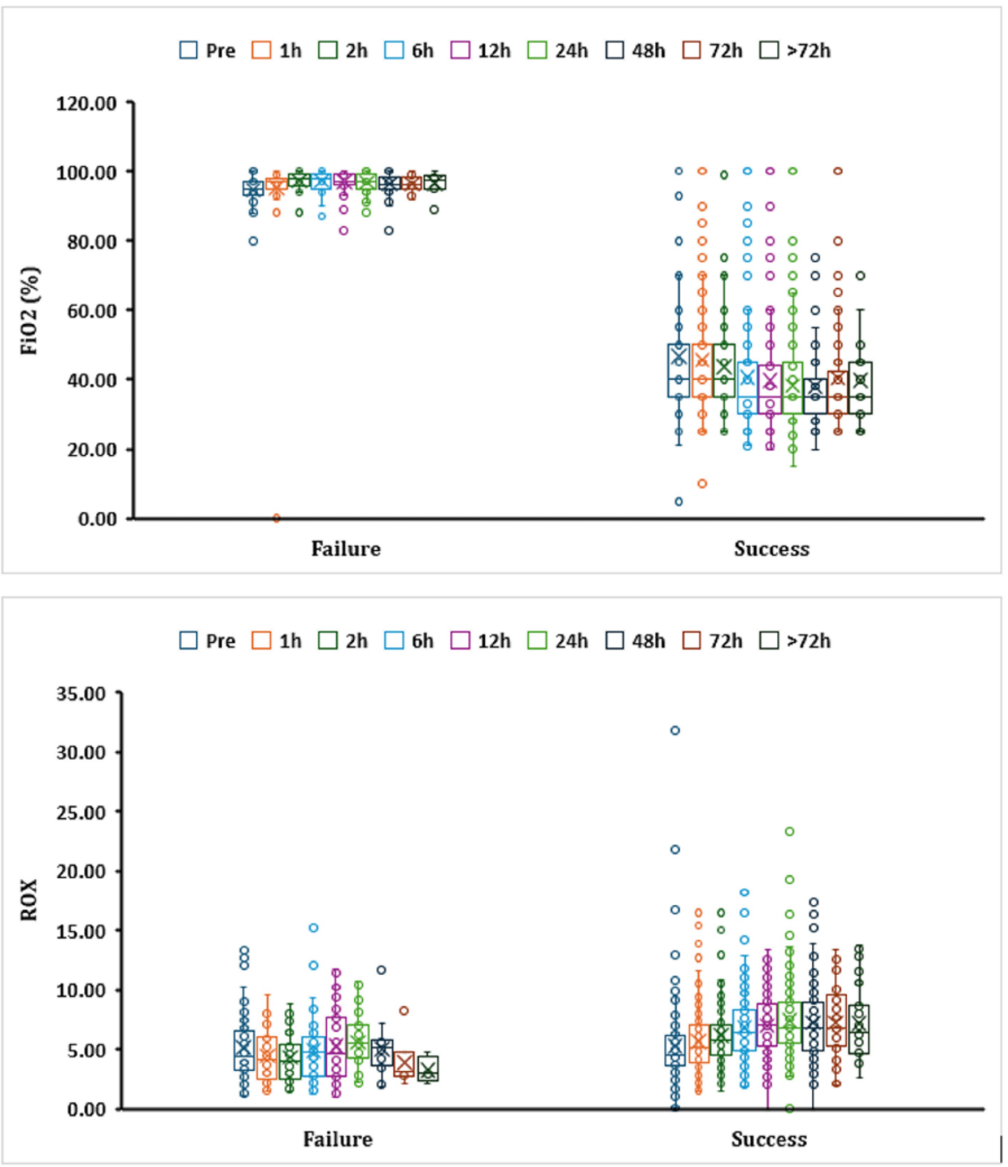
Box plot showing median FiO2 and ROX index for children who were on HFNC therapy against the outcome of HFNC therapy at different time intervals HFNC: high-flow nasal cannula; FiO_2_: fraction of inspired oxygen; ROX: residual oxygen consumption; h: hours

Higher ROX index values generally indicate a better response to HFNC therapy and a lower likelihood of needing intubation. Conversely, lower values suggest a higher risk of treatment failure and may prompt consideration of escalation of therapy. In the success group, ROX was always greater than 4.88, which means that they had a low risk of intubation (Figure 2).

At one, 72, and >72 hours, the ROX in the failure group was lower than 3.85, indicating a high risk of intubation, and in the success group, ROX was always greater than 4.88, which means that they had a low risk of intubation. However, from one hour till the end of HFNC intervention, the failure group showed marked elevations in FiO_2_, with significant declines in the ROX index, as compared to the success group.

**Figure 3:**
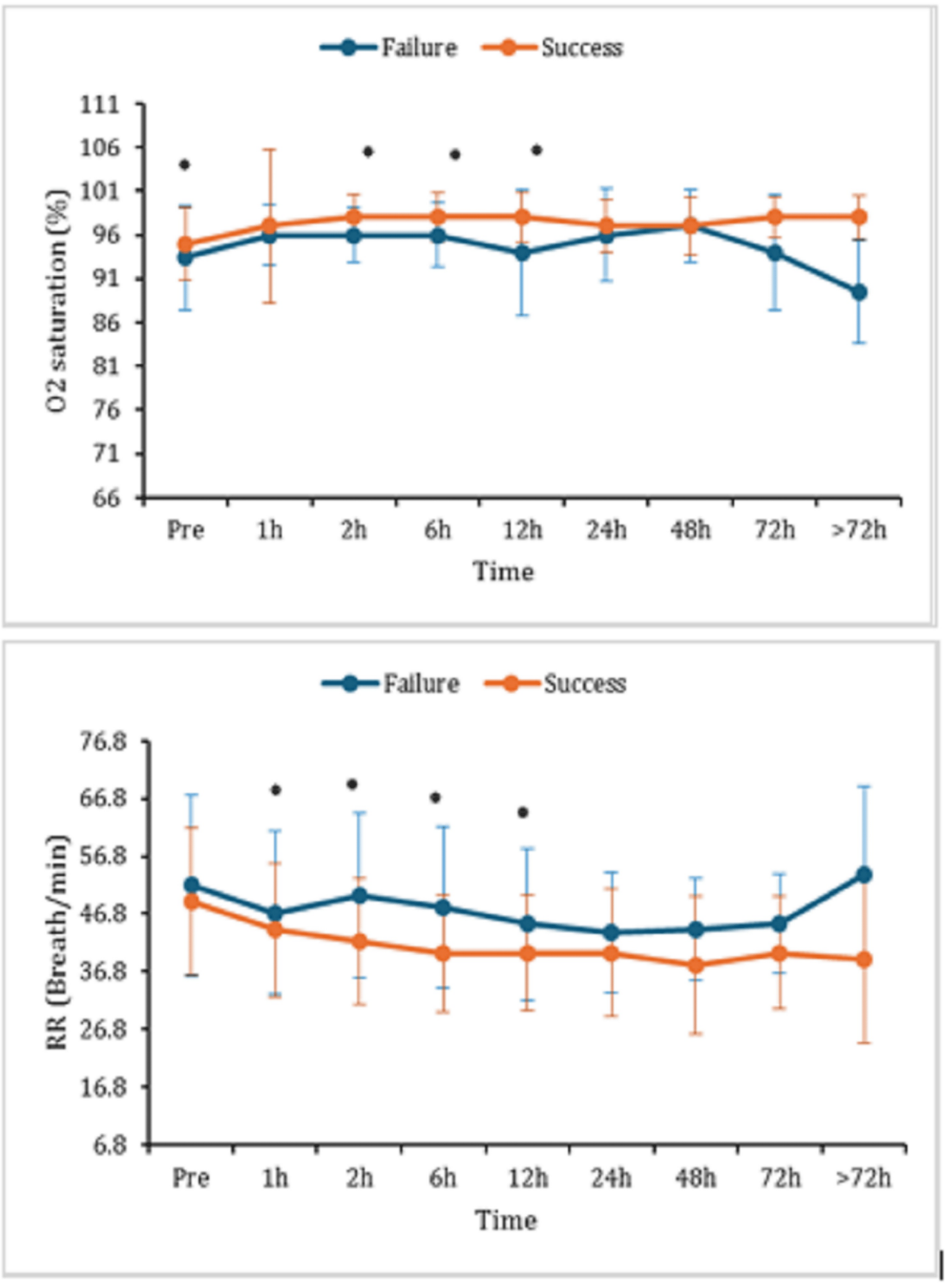
Percentage of oxygen saturation and respiratory rate at pre, one, two, six, 12, 24, 48, 72 hours, and >72 hours after the HFNC initiation. Data are displayed as mean (±SD); *: represents a significant (p < 0.05) difference between success and failure groups. HFNC: high-flow nasal cannula; O_2_: oxygen; RR: respiratory rate h: hours

As compared to the success group, oxygen saturation in the blood of the failure group was significantly reduced at two, six, and 12 hours after using HFNC. The respiratory rate was markedly elevated in the failure group than in the success group after one, two, six, and 12 hours of treatment with HFNC (Figure 3).

**Figure 4:**
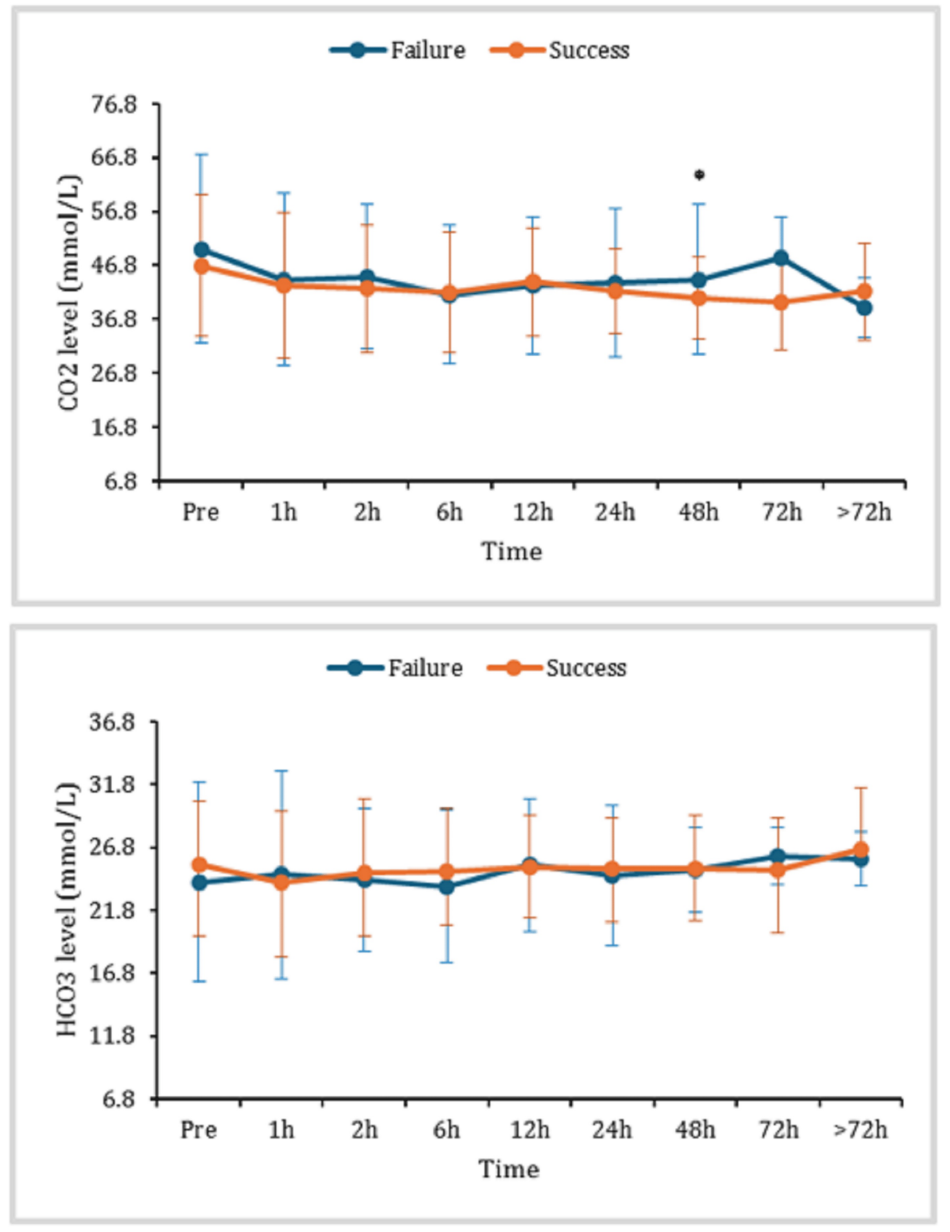
The blood levels of CO2 and HCO3 at pre, one, two, six, 12, 24, 48, 72 hours and >72 hours after the HFNC initiation. Data are displayed as mean (±SD); *: represents a significant (p < 0.05) difference between success and failure groups. HFNC: high-flow nasal cannula; CO2: the blood levels of carbon dioxide; HCO3: bicarbonates; h: hours

Blood levels of carbon dioxide (CO2_)_ and bicarbonate (HCO3) were monitored over time to assess the effectiveness of the intervention HFNC therapy (Figure 4). The results showed the failure group appeared to have higher CO2 levels compared to the success group throughout the monitored period. Statistical significance between the groups might be present after 48 hours only. The graph also shows no statistically significant differences in HCO3 levels between the two groups over the course of the study. While we observe slight variations in HCO3 levels across groups at each time point, these variations are consistent with typical physiological fluctuations.

Clinical and biochemical parameters were observed after the initiation of HFNC therapy: heart rate (HR), systolic blood pressure (SBP), and blood potential of hydrogen (pH). After one, two, and 48 hours of applying HFNC therapy. The HR was significantly higher in the failure group, as compared to the success group (Table 3).

**Table 3:** The HR, SBP, and PH in the blood at one, two, six, 12, 24, 48, 72 hours, and > 72 hours after the HFNC therapy initiation Data are displayed as mean (±SD); *: represents a significant (p < 0.05) difference between success and failure groups. HFNC: high-flow nasal cannula; HR: heart rate; SBP: systolic blood pressure; pH: potential of hydrogen; h: hours

Parameter	Time (hours)	HFNC outcome		p-value
		Success	Failure	
HR (count min-1)	Pre-HFNC therapy	139.6 ± 24.01	144.80 ± 22.67	0.103
	1	132.80 ± 20.75	139.10 ± 21.46*	0.033
	2	127.30 ± 18.75	137.90 ± 21.37*	0.007
	6	129.30 ± 21.43	135.30 ± 19.35	0.093
	12	125.70 ± 19.88	130.70 ± 20.08	0.146
	24	122.50 ± 20.09	127.30 ± 21.43	0.209
	48	119.90 ± 19.54	132.40 ± 26.49*	0.029
	72	116.70 ± 17.99	126.40 ± 15.74	0.185
	>72 h	119.10 ± 17.81	125.30 ± 4.99	0.502
SBP (mmHg)	Pre-HFNC therapy	99.05 ± 15.37	102.7 ± 21.24	0.127
	1	97.53 ± 14.88	99.18 ± 20.21	0.484
	2	97.69 ± 12.31	95.17 ± 19.92	0.384
	6	97.56 ± 13.58	101.50 ± 13.14	0.091
	12	97.24 ± 17.52	98.54 ± 15.03	0.652
	24	96.08 ± 13.05	97.53 ± 15.92	0.581
	48	97.94 ± 13.30	98.82 ± 13.88	0.805
	72	95.68 ± 12.38	105.90 ± 15.88	0.060
	>72h	95.28 ± 12.08	97.50 ± 15.00	0.743
pH	Pre-HFNC therapy	7.41 ± 0.07	7.32 ± 0.11*	0.002
	1	7.371 ± 0.13	7.34 ± 0.11	0.167
	2	7.382 ± 0.06	7.36 ±0.07	0.144
	6	7.401 ± 0.08	7.37 ±0.09	0.057
	12	7.406 ± 0.07	7.38 ±0.08	0.054
	24	7.401 ± 0.06	7.36 ± 0.09*	0.037
	48	7.409 ± 0.055	7.36 ± 0.08*	0.043
	72	7.417 ± 0.07	7.34 ± 0.05*	0.008
	>72 h	7.432 ± 0.055	7.43 ± 0.05	0.815

**Figure 5:**
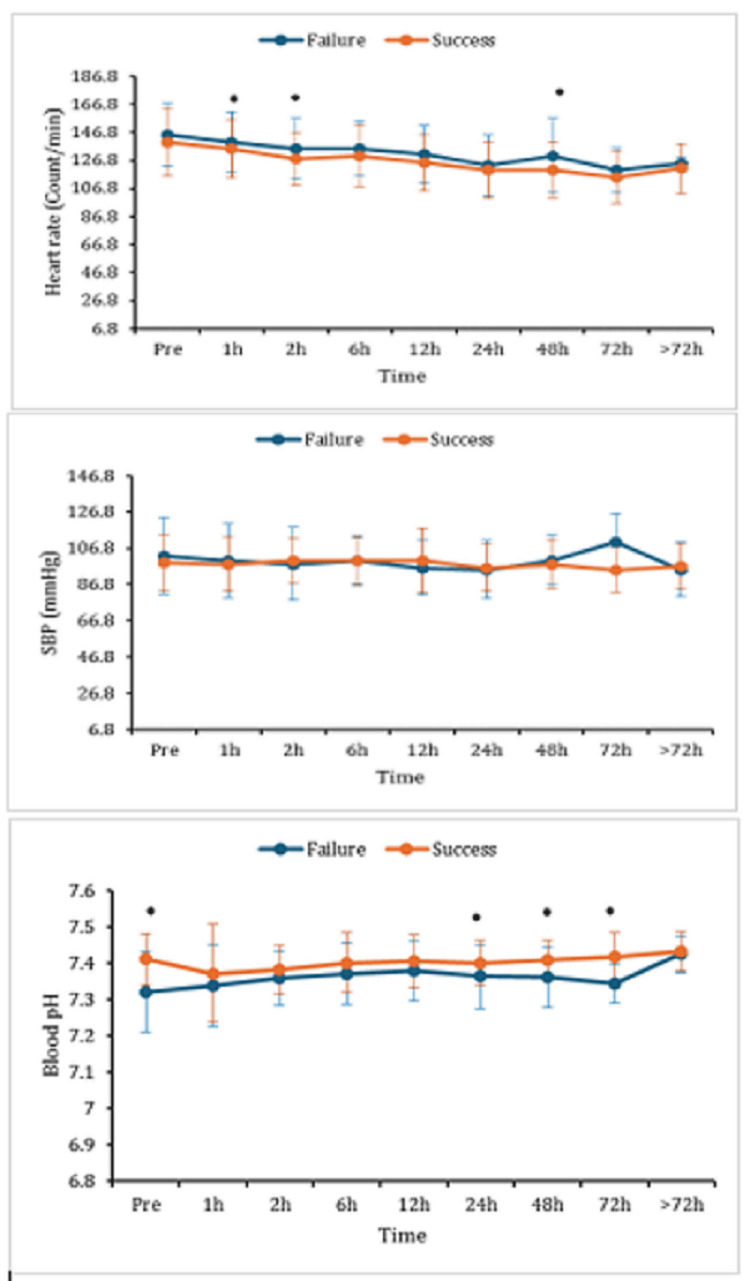
The HR, SBP, and pH in the blood at pre, one, two, six, 12, 24, 48, 72 hours, and >72 hours after HFNC therapy initiation. Data are displayed as mean (±SD); *: represents a significant (p < 0.05) difference between success and failure groups. HFNC: high-flow nasal cannula; HR: heart rate; SBP: systolic blood pressure; pH: potential of hydrogen; h: hours

No significant differences were recorded in SBP between success and failure groups. Blood pH in the failure group showed remarkable declines at 24, 48, and 72 hours after using HFNCs, as compared to the success group (Figure 5). 

## Discussion

Outcomes of HFNC therapy

The study of 265 patients treated with HFNC found no significant differences in age, gender, nationality, or BMI between the success and failure groups. The median age was nine months, with males making up 61.1% of the population. However, the duration of HFNC use significantly differed, with the success group requiring a median of 28 hours compared to 19 hours in the failure group (p < 0.001). Our study highlights key findings regarding the use of HFNCs in the PICU. High-flow nasal cannulas are commonly used for acute bronchiolitis, pneumonia, and post-extubation support, with intubation rates ranging from 5% to 43%. Predictors of HFNC failure include elevated respiratory rates, CO2 retention, and low pH. Complications are less frequent compared to CPAP and mechanical ventilation (MV), with nasal irritation being the most common. Viral infections, especially respiratory syncytial virus (RSV), significantly burden the PICU.

Indications and rate of failure of HFNC in the PICU

Acute bronchiolitis has been the main indication for HFNC use in the PICU. However, other indications have been investigated, including pneumonia, congenital heart disease, and post-extubation respiratory support. Habra et al. compared a different modality of noninvasive respiratory support and reported a higher failure rate of HFNCs compared with BiPAP or CPAP in the management of acute bronchiolitis in the PICU [5]. However, Coletti et al., with 620 children in their PICU settings, investigated various indications for the use of HFNCs, including status asthmatics (27.5%) and bronchiolitis (23.7%) [6].

Furthermore, they stated that 4.5% of cases needed a transition of therapy to intubation with MV, and 5.6% of cases needed escalation to NIV. Asseri et al. documented 92 cases of respiratory distress, with 40% of cases infected with bronchopneumonia and 13% with bronchiolitis; 23% of cases underwent tracheal intubation after the failure of HFNC therapy [7]. In addition, Baudin et al. investigated 177 cases, including more (52%) with congenital heart disease, 16% with bronchiolitis, and 7% with pneumonia. Baudin et al. stated that 22% of cases failed and required intubation with MV [8].

In our study, we investigated 265 cases, including 38.5% of cases with pneumonia, 13.2% with bronchiolitis, and 8.7% with asthma. Furthermore, 17% of cases required tracheal intubation, and 13.6% of them needed NIV. A review of the literature documented intubation rates of children between 5% and 43%, which agrees with the findings of our study [9].

Complications of HFNC in the PICU 

In the PICU, CPAP, and MV are essential respiratory support modalities but come with significant complications. Continuous positive airway pressure can cause nasal congestion, skin irritation, and pressure sores in pediatrics.

Patients, with younger children are particularly prone to discomfort and compliance issues due to the mask. Additionally, air leaks and aerophagia can lead to abdominal distension and discomfort. Mechanical ventilation in pediatric patients poses significant risks, including ventilator-associated pneumonia (VAP), barotrauma, and ventilator-induced lung injury (VILI). Prolonged MV use can result in ventilator dependence and muscle weakness, complicating the weaning process. Both therapies necessitate continuous monitoring and individualized adjustments to avoid these complications [10].

Conversely, HFNC provides better comfort and tolerance compared to traditional oxygen. Moreover, HFNC therapy is less invasive than MV, reducing the risk of ventilator-associated complications. Its ease of use and ability to provide continuous, adjustable flow rates for young patients. One small study investigated children's tolerance and found that children's comfort measured by the COMFORT scale improved after switching from oxygen delivered by a normal nasal cannula or face mask to HFNCs [11]. Baudin et al. reported complications with (1% and 3%) of pneumothorax and chest tube-related air leaks, respectively [8]. 

Vásquez-Hoyos et al. stated that 2.4% of cases experienced epistaxis, and four deaths out of 539 cases were documented in this study [12]. In addition, Sunkonkit et al. investigated 250 children and reported complications in only 10 of them, including one case of epistaxis, three cases of pressure sores, and six cases of gastric distension [13]. In our study, we documented only six cases of nasal irritation. Moreover, no epistaxis episodes or pneumothorax were detected.

Predictors of failure of HFNC in the PICU

We observed that age, diagnosis at admission, and positive viral nasopharyngeal aspirate (NPA) have no effect on the outcome of HFNC therapy. However, regarding the failure group in our study, respiratory rate was elevated pre-HFNC, oxygen saturation was greatly reduced, and also low durations were strongly associated with HFNC therapy failure. Furthermore, CO2 levels were higher pre-HFNC and after the initiation of HFNC therapy, and pH declined after the initiation of HFNC compared to the success group. Abboud et al. reported similar findings, stating that age did not influence response to HFNC; persistent desaturations after initiation of therapy were associated with therapy failure; pH was significantly lower both before and after initiation of therapy in the failure group, and partial pressure of carbon dioxide (PCO2) was the strongest indicator of HFNC therapy failure [9].

A review of literature about predictors of failure showed interesting insights, including Chang et al., who stated that age, sex, and primary indication for HFNC, such as pneumonia and bronchiolitis, didn't influence the success of therapy, but lower initial saturations may need escalation of therapy [14]. Vásquez-Hoyos et al. also reported that high respiratory rate is a significant risk factor for failure of HFNC therapy. Moreover, Betters et al. stated that high FiO_2_ requirement and history of intubation were risk factors for HFNC therapy failure [15].

We observed that a higher ROX index score was associated with HFNC therapy success and a low risk of intubation, which agrees with many studies [16] [14]. Meanwhile, Vasquez-Hoyos et al. reported that the ROX index may not be reliable for predicting HFNC therapy [17]. Moreover, Yuniar et al. stated that the ROX index is not useful as a predictor of HFNC in pediatrics but can only be useful at 60 minutes and 90 minutes with high sensitivity and specificity [18].

We observed that higher respiratory rates, carbon dioxide retention, and lower pH values are significant risk factors for HFNC failure. Additionally, patients with a history of intubation and high oxygen therapy requirements should be considered for alternative supplemental oxygen therapy options. Many clinicians suggested using BiPAP, CPAP, or tracheal intubation with the more critical children to avoid delay of the therapy in children with a higher risk of failing HFNC [19] [5].

Burden of viral infection in the PICU

Respiratory viral infections are among the most common causes of PICU admissions worldwide, especially in children under five years old, who are more susceptible due to their developing immune systems. For instance, RSV is a prominent cause of severe lower respiratory tract infections (LRTIs) in infants and young children, often leading to bronchiolitis and pneumonia [20]. During seasonal outbreaks, RSV alone accounts for up to 30% of PICU admissions [21]. Additionally, influenza can cause severe complications such as respiratory failure and is responsible for increased hospitalizations and ICU admissions during peak seasons [22]. The resource burden imposed by viral infections in PICUs is substantial, driven by the need for intensive monitoring, ventilation support, and prolonged hospital stays [23].

This study has its own limitations. For instance, it is a retrospective study conducted in a single center, which increases the likelihood of bias and limits the generalizability of the results. Patients who showed deterioration after only one hour of HFNC use were excluded. Furthermore, no scoring system was included to assess patient comorbidities or the severity of each disease.

## Conclusions

High-flow nasal cannula therapy is a safe and comfortable alternative to NIV and tracheal intubation, reducing intubation rates in the PICU. Lower pH, higher respiratory rates, and elevated PCO2 are predictors of HFNC therapy failure, while longer durations improve outcomes in conditions like pneumonia and bronchiolitis. Close monitoring is essential for critical cases, particularly those with cardiac comorbidities or high oxygen needs, to detect potential failure and complications.
